# Abstracts and Posters From the Annual Meeting of the Infectious Diseases Society for Obstetrics and Gynecology

**DOI:** 10.1155/S1064744996000695

**Published:** 1996


Abstracts and Posters From the

### Annual Meeting of the Infectious Diseases Society for Obstetrics and Gynecology

Las Croabas, Puerto Rico

August 6–9, 1997

**Reprinted from *Infectious Diseases in Obstetrics and Gynecology***
**Volume 4, Issue 6**

Infectious Diseases in Obstetrics and Gynecology 4:348–364 (1996)
© 1997 Wiley-Liss, Inc.

### ABSTRACT PRESENTATIONS

### INITIAL EXPERIENCE WITH DOUBLE-NUCLEOSIDE THERAPY FOR THE TREATMENT OF HIV INFECTION IN PREGNANT WOMEN: SAFETY PROFILES IN WOMEN AND NEWBORNS.

NS Silverman MD; MM DiVito RN, MSN; D Robbins MSN, CRNP. Division of MFM, Jefferson Medical College of Thomas Jefferson Univ., Phila. PA.

**Objective:** While treatment regimens combining two or three antiretrovirals have become standard for HIV-infected adults, concerns over perinatal safety have limited the widespread use of agents other than zidovudine (AZT) in pregnant women. While it has been suggested that maternal or neonatal side effects with newer nucleoside agents may not be significantly different from those reported in large-scale trials with AZT alone during pregnancy, no objective data from human pregnancies are available. We present our preliminary data from a policy offering AZT to all pregnant women, with the intent of decreasing vertical transmission in accordance with established guidelines, but that also offers a second nucleoside agent for maternal therapy, in accordance with current adult HIV protocols.

**Study Design:** Beginning in 1996, HIV-infected women at our institution received extensive individual counseling and were offered therapy with both AZT and a second nucleoside if they were either AZT-non-naive or had abnormal lymphocyte subset profiles at diagnosis. Hematologic, chemistry, and lymphocyte profiles were followed through their pregnancies, with blood counts and liver function testing performed on all newborns.

**Results:** From May 1996 to the present, nine women treated with double-nucleoside therapy have delivered; another two pregnancies are ongoing. AZT therapy (200 mg TID) was instituted or continued in women according to current guidelines at a median gestational age (GA) of 14 weeks (range 7–25), with two women already on therapy when pregnancy was diagnosed. A second nucleoside agent was prescribed in this cohort at a median GA of 24 weeks (range 14–37): two women early in the study period received didanosine (ddI), 250 mg BID; the others were treated with lamivudine (3TC), 150 mg BID. No patients stopped therapy for side effects and none had significant changes in hematologic or liver-function values from baseline to delivery. All patients delivered at term (mean GA 38.7 ± 1.1 wks), with a mean birthweight of 3182 ± 620 gms. In the newborns, 4/9 had mild anemia (HCT<50%), with a mean Hgb of 14.3 ± 4.7 gm/dl and Hct of 46 ± 8.8%; their mean white blood cell and platelet counts were normal. No newborns required blood transfusions, and all were discharged with their mothers after normal exams in the nursery. Elevated transminases were seen in 7/9 newborns (mean SGOT 56 ± 16 IU/l, SGPT 19 ± 9 IU/l), but none required therapy for hyperbilirubinemia.

**Conclusions:** Our experience with double-nucleoside therapy in this limited cohort of HIV-infected women provides early, objective support to its use during pregnancy for maternal indications. Effects on maternal and neonatal clinical status and lab profiles were comparable to those reported with AZT alone, and overall acceptance and tolerance were high.

### β-CHEMOKINE LEVELS IN PLASMA AND CERVICOVAGINAL LAVAGE (CVL) SPECIMENS FROM HIV1-INFECTED AND UNINFECTED WOMEN.

Antonio Sison, Marek Jasinski, Michael Spence, Joan Smith, Christian Hoffman, Danuta Kozbor

**Objective:** The β-chemokines MIP-1α, MIP-1β and RANTES have been shown to inhibit infection of CD4+ T cells by macrophage-tropic (M-tropic) HIV1 strains by blocking the β-chemokine receptor, CC-CKR-5, which is the principal cofactor for entry of M-tropic HIV1 isolates[f]. Individuals who show resistance to sexual transmission of HIV1 have T cells that cannot be infected *in vitro* by M-tropic HIV1[§], presumably because high endogenous levels of β-chemokines may block entry and infection. However, the site of action of these cytokines (vagina vs blood) has been poorly described. Our aim was to measure levels of β-chemokine production in plasma and CVL from infected and uninfected women to assess any protective role that these β-chemokines play in both sexual and perinatal transmission of virus.

**Study Design:** The cervicovaginal area of 18 asymptomatic HIV1-infected and 2 uninfected patients was lavaged with 10 mls of RPMI-1640. Heparinized plasma and aliquots of the CVL samples were then tested for levels of β-chemokines by quantitative ELISA, and reported as ρg/ml.

**Results:** (Standard deviations are given with each mean.)

| Patients with positive signals | RANTES | MIP-1α | | MIP-1β | |
|---|---|---|---|---|---|
| | CVL | PLASMA | CVL | PLASMA | CVL |
| HIV+ (n = 18) | 6 | 3 | 2 | 6 | 4 |
| Mean (ng/ml) | 524 ± 968 | 392 ± 295 | 984 ± 279 | 202 ± 121 | 194 ± 144 |
| HIV− (n = 2) | 2 | 0 | 0 | 1 | 0 |

Increased levels of β-chemokines were detected in 9 out of 18 CVL samples from infected women. One patient who recently seroconverted had increased levels in all 3 chemokines. Seven of 18 infected patients had high levels of MIP-1α and MIP-1β in plasma. Importantly, no correlation was found when comparing levels of β-chemokines in the plasma and CVL.

**Conclusions:** 1) β-chemokines were detected in CVL in about half of HIV-infected women and 40% in plasma, but high levels in CVL did not correlate to those in plasma, suggesting an independent chemokine activity in these compartments, and 2) recent seroconversion may be associated with high β-chemokine activity. Studies are in progress to evaluate the effectiveness of sexual transmission among those patients with elevated β-chemokine levels.

[f]Deng et al. *Nature* 1996;381:661; [§]Paxton et al., *Nature Med* 1996; 2:412.

### TGF-B GENE EXPRESSION IN INFECTED GESTATIONAL TISSUES: IMPLICATIONS FOR THE BALANCE BETWEEN PRO- AND ANTI-INFLAMMATORY CYTOKINES.

Leonid L. Reznikov, M.D., Ph.D.[1], Ronald S. Gibbs, M.D.[1], Robert S. McDuffie, M.D.[2], Joan Eskens M.S.[1], Kimberly K. Leslie, M.D.[1] Department of Obstetrics and Gynecology, the University of Colorado Health Sciences Center[1] and Kaiser Permanente[2], Denver, CO

**Objectives:** In the rabbit model of chorioamnionitis and infection-induced preterm pregnancy loss, we have shown that amniotic fluid

TNF-α rises significantly with infection; however, the protein concentration and bioactivity of the anti-inflammatory cytokine TGF-β do not change. We speculate that rising TNF-α in the face of unchanging TGF-β creates an imbalance between pro- and anti-inflammatory cytokines which favors prostaglandin production. We now report an analysis of TGF-β gene expression in control versus infected pregnant uterine tissues. The goal of these studies is to determine whether mRNA levels correlate with TGF-β protein levels and bioactivity.

**Study Design:** New Zealand white rabbits at 70% gestation underwent hysteroscopic inoculation of $10^5$ cfu E. coli or saline (placebo) and were sacrificed 16 h later. Total RNA was isolated and reverse transcribed (RT). cDNA fragments for TGF-β 1, TGF-β 2, and β-actin were amplified by the the polymerase chain reaction (PCR), and the products were run on ethidium bromide-stained agarose gels. The specific bands for each amplified fragment were analyzed by densitometer, and the relative expressions of TGF-β 1 and 2 were determined compared to β-actin for infected and control animals. The results were semi-quantitative, and expressed as the percent of densitometric units of TGF-β compared to β-actin.

**Results:** No gross differences in TGF-β 1 or 2 mRNA levels were detected by semi-quantitative RT-PCR analysis of the decidua and full-thickness uterine wall in infected pregnant rabbits versus controls.

**Conclusion:** Consistent with our previous measurements of TGF-β protein concentrations and bioactivity in the amniotic fluid of infected versus control pregnant rabbits, our analysis of TGF-β gene expression demonstrates no significant induction of TGF-β messages with infection. The lack of compensatory rise in TGF-β with infection in our pregnant model suggests that TGF-β gene transcription is differentially regulated compared to other infectious and inflammatory conditions, where TGF-β expression is significantly up-regulated.

**CERVICOVAGINAL CYTOKINES IN PREGNANT WOMEN WITH BACTERIAL VAGINOSIS AND FOR TRICHOMONIASIS.** Begona Martinez de Tejada, Sharon L. Hillier, Dan V. Landers, Department of Obstetrics, Gynecology, and Reproductive Sciences, University of Pittsburgh and Magee-Womens Research Institute, Pittsburgh, PA.

**Objectives:** To evaluate the association between cervicovaginal interleukin 1β (IL-1β), interleukin 8 (IL-8), and interleukin 6 (IL-6) with bacterial vaginosis (BV) and trichomoniasis.

**Study Design:** Cross-sectional study of 311 women at less than 20 completed weeks' gestation. BV was defined as pH >4.5 and Gram stain score ≥ 7 (Nugent criteria). Trichomoniasis (TV) was diagnosed by broth culture.

**Results:** The median levels of the three cytokines stratified by infection is shown below.

| | | Cytokine (median pg/ml) | | |
|---|---|---|---|---|
| Infection | N | IL-1β | IL-6 | IL-8 |
| BV + TV+ | 14 | 5240* | 516 | 62,108* |
| BV + TV− | 116 | 4407* | 320 | 13,726* |
| BV − TV+ | 12 | 7682* | 345 | 52,687* |
| BV − TV− | 169 | 1532 | 370 | 19,390 |

*P < 0.05 Mann-Whitney U Test

Pregnant women with either TV or BV have significantly increased concentrations of IL-1β comparedto uninfected women. IL-8 was significantly *increased* in women with TV, and significantly *decreased* in women with BV, compared to women with no infection. There was no association between elevated IL-6 and BV or TV.

**Conclusions:** These data suggest that both BV and TV detected in the first half of pregnancy are associated with increased levels of IL-1β. The IL-8 findings may reflect the contribution of PMN's in TV infection and possible downregulation of PMN recruitment by BV pathogens or their metabolic products.

*Young Investigator Award*

**HIGH VAGINAL INTERLEUKIN-8 LEVELS AMONG WOMEN IN PRETERM LABOR ARE ASSOCIATED WITH PRETERM DELIVERY.** J Hitti, SL Hillier, MA Krohn, DA Eschenbach; Department of Obstetrics/Gynecology, University of Washington.

**Objective:** To study the associations between vaginal flora, interleukins (IL); and preterm delivery.

**Methods:** 214 women in preterm labor with intact membranes at 22–34 weeks gestation had vaginal samples collected for Gram stain, culture, IL-6 and IL-8 determinations by EIA. The distributions of IL-6 and IL-8 were evaluated using the Mann-Whitney test. Risk ratios for preterm delivery were calculated for high vaginal IL-8 in association with normal or disrupted vaginal flora by Gram stain.

**Results:** Vaginal IL-8 levels were higher among patients delivering preterm (p < 0.001):

| Delivery at ≤ 34 Weeks | N | IL-8 (pg/ml) Median (95% CI) | IL-6 (pg/ml) Median (95% CI) |
|---|---|---|---|
| Yes | 107 | 40,960 (21,380 − 77,440) | 1,720 (<700 − 3,260) |
| No | 107 | 10,240 (5,260 − 16,630) | 1,660 (<700 − 2,520) |

Vaginal IL-8 levels were significantly higher among patients with vaginal isolates of *M. hominis* (p < 0.01), >$10^6$ *G. vaginalis* or >$10^4$ black *Prevotella* (p < 0.05), but were not significantly higher among patients with abnormal vaginal flora by Gram stain. High vaginal IL-8 levels (>75,000 pg/ml) were associated with preterm delivery independent of vaginal Gram stain characteristics.

| | N | Delivery at ≤ 34 Weeks | RR (95% CI) |
|---|---|---|---|
| Vaginal IL-8 ≤ 75,000 pg/ml | | | |
| Normal vaginal flora by Gram stain | 94 | 32 (34%) | reference |
| Bacterial vaginosis/ intermediate flora | 66 | 35 (53%) | 1.6 (1.1 − 2.2) |
| Vaginal IL-8 > 75,000 pg/ml | | | |
| Normal vaginal flora | 30 | 21 (70%) | 2.1 (1.4 − 3.0) |
| Bacterial vaginosis/ intermediate flora | 24 | 19 (79%) | 2.3 (1.6 − 3.3) |

**Conclusions:** Among women in preterm labor, high vaginal IL-8 may be an independent risk factor for preterm delivery. The relationships between abnormal vaginal flora and vaginal IL-8 require further study.

**THE ROLE OF *dra* OPERON AND NITRIC OXIDE IN *ESCHERICHIA COLI* UTERINE INFECTION IN PREGNANT RATS.** B Nowicki, MD, PhD[1,2] L Fang, MD[1] J. Singhal,[1] S Nowicki, DDS[1,2] C Yallampalli, DVM, PhD.[1] Departments of OB/Gyn[1], & Microbiology[2], University of Texas Medical Branch, Galveston, TX

**Objectives:** The mechanism of urogenital infections caused by *E. coli* and associated complications during pregnancy is not well understood. Nitric oxide (NO), an endogenous smooth muscle relaxant involved in maintaining uterine quiescence during pregnancy is considered to participate in an anti-bacterial mechanism. In this study, we evaluated the effects of mutation of *dra* operon encoding *E. coli* Dr fimbriae and NO inhibitor L-NAME on uterine infection in pregnant and nonpregnant animals.

**Study Design:** Rats implanted with osmotic minipumps with either L-NAME or saline only were inoculated with Dr fimbriae positive (Dr+) *E. coli* or its mutant (Dr–) (200 μl $5 \times 10^9$ bacterial cells/ml) into the cavity of the left uterine horn (72 rats on day 18 of pregnancy and to 12 nonpregnant). Animals were sacrificed on day 19,20,21 (pregnant) or at 1 and 2 days after infection (nonpregnant). Rats were monitored for the death rate and preterm labor; kidney, spleen, liver, uterus, placenta, fetuses, and blood were collected for quantitative cultures.

**Results:** Within 24 hours after infection, 31% of pregnant animals were dead in Dr+ group only. Interestingly, co-treatment with L-NAME resulted in two-fold increase in death rate (66%) in Dr+ group. No deaths occurred in nonpregnant rats either with or without L-NAME. Infection of the uterus in pregnant animals was $5 \times 10^5$/g in the Dr+; $5 \times 10^4$/g in Dr– group. In pregnant rats, Dr+ *E. coli* in the blood were detectable in 75% of the untreated and 88% of the L-NAME treated group. The dissemination of infection to the kidney, spleen, and liver was $8 \times 10^6$ bacteria per gram of tissue with Dr+ *E. coli* and this was about 3 logs lower ($p \leq 0.01$) with Dr– *E. coli.* Controls without infection, with or without L-NAME were unaffected in terms of death or infection spread. No preterm labor was observed in any pregnant animals.

**Conclusions:** Dr+ *E. coli* displayed significantly higher virulence to pregnant rats only. Inhibition of NO enhanced the dissemination of infection and death in the pregnant animals infected with Dr+ but not Dr– *E. coli.* We therefore propose that infectious complications of pregnancy may be related to both 1) gestation-dependent virulence of the pathogen, and 2) the host sensitivity associated with the NO status.

***E. COLI* ACQUISITION DURING PREGNANCY AND RISK OF PRETERM BIRTH.** HM McDonald[1] PhD, JA O'Loughlin[1] FRACOG, R Vigneswaran[1] FRACP, PJ McDonald, FRACP, A Bof[2] MBBS, MF Annells[1] MHSM, PT Jolley[1] B Pharm, J Kennedy[2]. [1]Women's & Children's Hospital, [2]Lyell McEwin Health Service, Adelaide, Australia.

**Objectives:** Women with preterm birth (PTB) <34 weeks have a nearly 4-fold higher prevalence of *E. coli* in labour. The purpose of this study was to determine the acquisition/carriage of *E. coli* serogroups in the urine and vagina during pregnancy, and relationship to PTB.

**Study Design:** Pregnant women with a history of PTB (PPTB) or of urinary tract infection (UTI), and a control group of women with no history of PTB or UTI, were enrolled. Cultures of vaginal swabs (semi-quantitative) and urine (quantitative) were performed at 24, 28, 32, and 36 weeks' gestation, and in early labour. Women with age <17 years were excluded.

**Results:** 474 women were enrolled (151 UTI, 81 PPTB and 242 controls). The demographic characteristics of the three groups were similar. The spontaneous PTB rates were: 2.1%, 5.4% and 22.1% for the control, UTI and PPTB groups. There was little site variation in the *E. coli* prevalence/visit in controls (14.9% vagina, 14.7% urine), although this was greater in PPTB women (18.6%, 14.5%). Although half the control (49.2%) and PPTB (48.1%) women had *E. coli* in the vagina/urine at some stage during pregnancy, 61.6% of women in the UTI group had *E. coli* during pregnancy [OR 1.66 (95%CI 1.07–2.56)]. Prevalence increased with advancing gestation in the UTI and PPTB groups but acquisition during late pregnancy or early labour was rare. The most common serogroups were O1, O6, O75, O68 and O16, and two thirds of women with *E. coli* had only one serogroup during pregnancy. O1 was significantly more common in the UTI group than in controls [19.2% vs. 10.3% OR 2.06 (1.11–3.83)] or PPTB. O6 was the most persistent serogroup overall. O16 was significantly more common in PPTB women than in the control [9.9% vs. 2.9%, OR 3.7, (1.1–12.3)] or UTI [2.0%, OR 5.4 (1.3–32.3)] groups. In the 8 PPTB women with O16, a 50% PTB rate (<37 weeks) was found vs. 18.8% (13/69) in the remainder of the PPTB group.

**Conclusions:** Women with PPTB have an increased vaginal/urine prevalence of *E. coli* O16 compared with women with UTI or controls (despite a higher overall *E. coli* colonisation in women with UTI). Women with *E. coli* O16 and PPTB may have a higher PTB rate than other PPTB women, but this is not associated with late acquisition.

**HIGH DENSITY GENITAL GROUP B STREPTOCOCCAL COLONIZATION INCREASES THE RISK OF INTRA-AMNIOTIC INFECTION.** Marijane A. Krohn, Ph.D., Sharon L. Hillier, Ph.D., Carol J. Baker, M.D.

**Objectives:** To determine the risk of intra-amniotic infection (IAI) associated with high density maternal vaginal group B streptococci (GBS) colonization.

**Study Design:** A cross-sectional cohort of 5587 women was enrolled from 1992 to 1996 in three cities: Houston TX, Seattle WA, Pittsburgh PA. IAI was defined as fever of 38°C accompanied by two of the following signs: maternal (≥100 bpm) or fetal (≥160 bpm) tachycardia, uterine tenderness, purulent amniotic fluid, and elevated peripheral WBC count (>15,000). Vaginal specimens obtained at admission for delivery were inoculated into a selective enrichment broth and blood agar. Agar plates were streaked in four zones. High density was defined as growth in zones 3–4 and low density in zones 1 or 2.

**Results:** The frequency of IAI was: Houston, 2.5% (18/712); Seattle, 5.3% (145/2755); Pittsburgh, 0.9% (20/2121). High density GBS colonization was present in 5% (289/5587), low density in 11% (620/5587), and broth only in 5.4% (302/5587) of women. After adjusting for city, high density colonization was associated with an increased risk of IAI (aRelative Risk (RR) = 1.85; 95% Confidence Interval (CI) = 1.00–3.25), low density colonization was associated with a minimally increased risk (aRR = 1.18; 95% CI = 0.76–1.80), and broth only was not associated with an increased risk (aRR = 0.70; 95% CI = 0.33–1.47).

**Conclusions:** These findings provide further evidence that intrapartum GBS colonization is associated with maternal complications. Screening and treatment intervention strategies are necessary to address IAI, as well as, other previously described complications of group B streptococcal genital colonization.

**THE EFFECT OF INTRAMUSCULAR BENZATHINE PENICILLIN G ON GROUP B *STREPTOCOCCUS* AND OTHER VAGINAL FLORA IN PREGNANT WOMEN.** Brock BV, Watts DH, Brown ZA, Agnew KJ, Eschenbach DA. University of Washington, Seattle, WA.

**Objective:** To evaluate the efficacy of intramuscular Benzathine Penicillin G (PCN) on suppression of vaginal Group B *Streptococ-*

*cus* (GBS) colonization in pregnant women and to assess the impact of therapy on other vaginal flora.

**Study Design:** Pregnant women with GBS colonization were recruited from High Risk and Diabetes clinics. Vaginal specimens for gram stain, semi-quantitative culture for GBS, enteric gram negative rods, *Enterococcus* and yeast were obtained at baseline and every one to two weeks after I.M. administration of 2.4 million units of PCN and upon admission in labor. All patients who labored received intrapartum I.V. antibiotic GBS prophylaxis. Cultures were obtained from between the chorion and amnion at delivery.

**Results:** 17 patients with GBS colonization received I.M. PCN. 14 patients became GBS culture negative in a median of 7 days (range 4–40) after one injection. Three patients required multiple injections to become GBS negative. Time to first negative culture was not related to level of colonization at baseline. Patients remained GBS negative a median of 32 days after the first PCN injection (range 14–81) and >21 days after repeat injections (range 14–55+). One (6%) of the 17 patients was GBS positive at delivery. Changes in vaginal flora are summarized below:

| | Baseline culture positive | Baseline negative, becomes positive | Baseline negative, positive at delivery | Total positive at delivery |
|---|---|---|---|---|
| GNR | 4/17 | 11/13 | 8/11 | 10/17 |
| *Enterococcus* | 1/17 | 12/16 | 6/12 | 6/17 |
| Yeast | 4/17 | 4/13 | 2/4 | 2/17 |

The majority of patients with GNR, *Enterococcus* and yeast at delivery are those who acquired these isolates after PCN administration. Nine out of 10 women with normal baseline gram stains remained normal at delivery, while 4 out of 7 with abnormal gram stains improved. Two (12.5%) of 16 chorioamnion cultures were positive (1-*E. coli*, 1-*Gardnerella vaginalis*). None of the 17 infants had neonatal infections.

**Conclusion:** Intramuscular PCN suppressed vaginal GBS colonization in a majority of patients. This strategy to prevent neonatal colonization at term needs to be studied in a larger group, however, the increase in vaginal colonization with GNR and *Enterococcus* could increase maternal and neonatal infections from these organisms.

### *STREPTOCOCCUS AGALACTIAE* (GROUP B STREP) RESISTANCE TO CLINDAMYCIN AND ERYTHROMYCIN.

Mark Pearlman, MD, Carl Pierson, PhD., Roger Faix, MD.

**Objectives:** Current recommendations for Group B strep (GBS) intrapartum prophylaxis in penicillin allergic patients is the use of either clindamycin or erythromycin. This study was designed to test the frequency of resistance of GBS clinical isolates to these and other frequently used antibiotics.

**Study Design:** One hundred isolates of Group B Strep collected from the lower genital tract/anorectum of 100 pregnant women were utilized for testing. Resistance to clindamycin (100 isolates), erythromycin (82 isolates), benzyl penicillin (82 isolates), cephalothin (82 isolates) and vancomycin (82 isolates) was tested using E-test strips on Mueller Hinton agar supplemented with 5% sheep RBCs incubated in a non-$CO_2$ atmosphere for 18 to 20 hours.

**Results:**

Most isolates resistant to clindamycin were also resistant to erythromycin; however, three isolates were resistant to erythromycin but

| | | No. (%) resistant to: | |
|---|---|---|---|
| Set No. | No. tested | Clindamycin | Erythromycin |
| 1 | 18 | 5 (28) | ND |
| 2 | 18 | 3 (17) | 5 (28) |
| 3 | 36 | 4 (11) | 4 (11) |
| 4 | 28 | 4 (11) | 4 (11) |
| Total | 100 | 15 (15) | 12/82 (15) |

sensitive to clindamycin and one isolate sensitive to erythromycin was resistant to clindamycin. All 82 isolates tested were sensitive to penicillin, cephalothin and vancomycin.

**Conclusions:** *In vitro* resistance of GBS to clindamycin and erythromycin occurs in a significant number of clinical isolates. Larger studies are necessary to confirm these rates of *in vitro* resistance, to assess the resultant impact on *in vivo* efficacy, and to determine if alternative antibiotics are more appropriate in pencillin allergic patients. Meanwhile, care providers should be aware of the possibility of clinical resistance to clindamycin and erythromycin and need to carefully review the history of penicillin allergy to ascertain if non-penicillin, non-cephalosporin alternatives are truly indicated.

### DETECTION OF BACTERIA IN AMNIOTIC FLUID BY A MONOCLONAL ANTIBODY TO AN EPITOPE OF THE ELONGATION FACTOR TU.

Penny Clark, Ph.D.,[1] Gregory Locksmith, M.D.,[1] Jörg Köhl, M.D.[2] and Patrick Duff, M.D. Dept. of Obstetrics and Gynecology, University of Florida[1] and Dept. of Medical Microbiology, Medical School Hannover, Hannover, Germany.[2]

**Introduction:** Elongation factor Tu (EF-Tu) catalyzes the binding of each amniocyl t-RNA to the ribosome. EF-Tu is one of the most abundant proteins in procaryotes, representing 5% of the total cellular protein of *E. coli.* MAb 900, a monoclonal antibody against a highly conserved epitope on the *E. coli* EF-Tu protein, has been found to have broad cross-reactivity with more than 90 species of bacteria.

**Objective:** To determine if MAb 900 will detect bacteria in amniotic fluid. If so, an assay using MAb 900 may be useful in the diagnosis of bacterial infection of the amniotic cavity.

**Study Design:** MAb 900, generated by immunization of mice with *E. coli K-12 C600,* was used for the assay of EF-Tu by ELISA. The antibody was tested on several species of aerobes and anaerobes commonly isolated from the female genital tract. EF-Tu assay was performed on amniotic fluid (AF) samples that were intentionally inoculated with known concentrations of pure cultures of *E. coli, Lactobacillus sp., Bacteroides fragilis, Peptostreptococcus,* and group B streptococcus to determine the assay's limit of detection. Lysates of *E. coli* obtained by sonicating whole cells for 2 minutes in buffer with glass beads were used as standard. A separate set of 40 AF samples were then collected from term (n = 20) and preterm (n = 20) pregnancies, cultured for bacteria, and assayed for EF-Tu. The EF-Tu assay results were compared to culture results.

**Results:** Amniocytes, RBCs and WBCs in amniotic fluid did not react to MAb 900, while the species of aerobic and anaerobic bacteria commonly isolated from the female genital tract showed strongly positive reactions. The assay by ELISA detected bacterial concentrations greater than $10^4$ bacteria/mL, and took 8 hours to perform. Of the 40 test AF samples, 4 had positive cultures. All 36 samples with negative cultures had a negative EF-Tu assay. Of the 4 samples with positive cultures, 2 were detected by the EF-Tu assay. The 2 samples that were not detected contained less than $10^2$ bacteria/mL.

**Conclusion:** The monoclonal antibody to a bacterial elongation factor identifies bacteria in amniotic fluid. At its present level of sensitivity ($10^4$ col./mL), the assay is of greatest value in excluding bacterial infection of amniotic fluid.

### *TRICHOMONAS VAGINALIS* BUT NOT BACTERIAL VAGINOSIS IS ASSOCIATED WITH INCREASED VAGINAL FLUID DEFENSINS IN PREGNANCY.

RP Heine, MD, LF Mortimer, MS, DL Draper, PhD, HC Wiesenfeld, MD, CM, MA Krohn, PhD. Magee-Womens Research Institute, Department Obstetrics Gynecology and Reproductive Sciences, University of Pittsburgh School of Medicine, Pittsburgh, PA.

**Objective:** *T. vaginalis* (TV) and bacterial vaginosis (BV) are associated with adverse pregnancy outcome. The mechanism may involve an interaction between the pathogen and the host immune system. We hypothesize that women with an exaggerated immune response in concordance with specific pathogens have a high risk of preterm birth. To investigate this hypothesis we initially sought to determine if neutrophil-specific defensins are elevated in patients infected with TV or BV.

**Study Design:** From 7/95 to 1/97, we screened all asymptomatic pregnant women entering prenatal care at Magee-Womens Hospital for TV by culture and BV by gram stain at 8–21 weeks gestation. An additional swab was obtained from the posterior fornix and frozen at −70°C for subsequent defensin analysis. Defensin levels were measured in batch by ELISA. Comparisons between groups were made by the Mann-Whitney U Test for non-parametric data.

**Results:** For this study we evaluated 64 women with TV, 60 with BV, and 62 controls. Patients with TV but not BV had significant elevations in defensins when compared to controls ($p < .005$). All groups exhibited a wide range of defensin levels.

**Conclusion:** Defensin levels are elevated in pregnant women infected with TV but not BV. The highly variable levels of elevation suggest differing degrees of neutrophil activation. Future work will focus on the degree of defensin elevation and correlation with adverse pregnancy outcome.

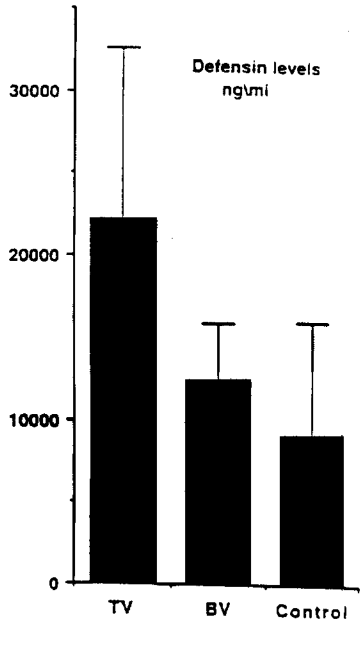

### THE UTILITY OF READILY AVAILABLE CLINICAL TESTS IN PREDICTING PELVIC INFLAMMATORY DISEASE IN WOMEN PRESENTING WITH PELVIC PAIN.

Soper DE, Peipert J, Ness R, Bass D, Hemsell D, Shepherd S, and the PEACH Study Group.

**Objectives:** To determine which tests were sufficiently predictive as to exclude the diagnosis of PID among women with the minimum clinical criteria (abdominal pain and pelvic organ tenderness).

**Study Design:** Reproductive age group women with a complaint of lower abdominal pain and uterine and adnexal tenderness were prospectively enrolled in a randomized clinical trial comparing inpatient and outpatient antibiotic therapy for PID (Group 1). After initiation of the study, additional inclusion criteria [presence of leukorrhea (vaginal wbc > epithelial cells), mucopurulent cervicitis and/or positive tests of the cervix for GC or chlamydia] were added (Group 2). Demographic and clinical variables were assessed in each group and the incidence of PID (defined as histologic endometritis and/or a positive test for gonorrhea or chlamydia from the endometrium) was compared. Sensitivity, specificity and positive and negative predictive values were determined for the following variables in Group 1; leukorrhea, mucopurulent cervicitis, bacterial vaginosis, any vaginal wbc, peripheral wbc count (>10,000), and ESR (>15).

**Results:** 63 women were enrolled in Group 1 and 174 women were enrolled in Group 2. There were no significant differences between Groups 1 and 2 with respect to demographic variables. The incidence of PID was 38.1% (24/63) in Group 1 and 48.9% (85/174) in Group 2 [$p = 0.14$]. The diagnostic indices for Group 1 were as follows:

| | Leukorrhea | MPC | BV | Vaginal wbc | Peripheral wbc | ESR |
|---|---|---|---|---|---|---|
| Sensitivity | 51.2% | 68.2% | 41.7% | 95.5% | 55.0% | 60.9% |
| Specificity | 70.3% | 91.7% | 73.0% | 21.1% | 80.0% | 64.1% |
| PPV | 51.2% | 83.3% | 50.0% | 41.2% | 61.1% | 50.0% |
| NPV | 70.3% | 82.5% | 65.9% | 88.9% | 75.7% | 73.5% |

**Conclusions:** The evaluation of the lower genital tract for evidence of inflammation is helpful in the diagnosis of PID. The absence of vaginal white blood cells during microscopy of the vaginal secretions is the most reliable test that rules out PID.

### ACTIONS AND ATTITUDES REGARDING HIV TESTING IN MINORITY WOMEN WITH STDS.

J.M. Piper, M.D., J.H. Dimmitt, Ph.D., R.N. Shain, Ph.D., E.R. Newton, M.D., S.T. Perdue, M.D.

**Objective:** Although universal screening for HIV in pregnancy is becoming widely accepted, attitudes regarding HIV risk and acceptance of HIV testing remain important issues, particularly in the women at highest risk—low socioeconomic status, minority women with other STDs. We sought to examine attitudes toward and acceptance of HIV testing in a low income, minority population with active STDs.

**Study Design:** Black and Hispanic women with an active STD underwent detailed questioning regarding STD beliefs and risk behaviors, including those related to HIV. Perception of HIV risk, HIV knowledge, risk behaviors, and attitudes regarding HIV testing were assessed, and compared between pregnant and nonpregnant women. HIV testing was offered to all women after the interview.

| Attitude/Action | Overall | Pregnant vs. Non-pregnant | P |
|---|---|---|---|
| Denied HIV Risk | 52% | 60% vs 49% | <0.02 |
| Did Not Know How HIV Is Spread | 6% | 9% vs 4% | <0.05 |
| No Prior HIV Test | 46% | 55% vs 42% | <0.005 |
| Tested, But Never Got Results | 26% | 26% vs 26% | NS |
| Continued to Refuse Testing | 9% | 9% vs 9% | NS |

None of the women were found to be HIV positive.

**Results:** 195 Black and 425 Hispanic women with an active STD were interviewed, of whom 184 (30%) were pregnant. Current STDs included gonorrhea (21%), chlamydia (69%), and trichomonas (23%).
**Conclusions:** Low perception of HIV risk is a significant impediment to voluntary HIV testing, even in women at high risk. Strategies to encourage HIV testing and reduce HIV transmission must focus on both reducing risk behavior and improving perception of HIV risk, particularly in high-risk populations.

### EFFECTS OF CHLORHEXIDINE GEL AND NONOXYNOL-9 ON VAGINAL FLORA AND EPITHELIUM.

DH Watts, L Rabe, MA Krohn, J Aura, SL Hillier. University of Washington, Seattle, WA and University of Pittsburgh/Magee-Womens Research Institute, Pittsburgh, PA.

**Objective:** To evaluate the effects of a single dose of chlorhexidine gluconate (CHG) gel or nonoxynol-9 (N-9) gel on vaginal flora and epithelium.
**Study Design:** Women underwent history, physical and colposcopic examination, vaginal Gram stain, and quantitative vaginal cultures at baseline and 30′, 4°, and 1, 2, 3, and 7 days after instillation of 2 ml of 0.05% CHG gel (Surgilube®) (n = 17) or one applicatorful of 4% N-9 gel (Conceptrol®) (n = 8).
**Results:** No changes in the vaginal epithelium were noted by colposcopy after CHG or N-9. After CHG, concentrations of $H_2O_2$+ and $H_2O_2$− lactobacilli (LB), and *Enterococcus* decreased by 0.8–1.1 log at 30′ and 4°, but returned to baseline by 24°. The one subject with group B *Streptococcus* at baseline became GBS negative thru day 2, but was positive again on day 3. Vaginal Gram stain results did not change significantly after CHG. After Conceptrol instillation, concentrations of $H_2O_2$+ LB decreased by 1–2 log at 30′ and 4° but returned to baseline by 24° while $H_2O_2$− LB levels did not change. Concentrations of *Enterococcus,* anaerobic Gram negative rods, and *G. vaginalis* decreased also at 30′ and 4°. Vaginal Gram stain scores increased on days 2 and 3, then returned to baseline. Three women acquired *E. coli* after N-9 instillation, and the subject with *E. coli* at baseline had a 3–4 log increase in level at 4 and 24°.
**Conclusions:** Single doses of intravaginal CHG and N-9 were well tolerated. Both CHG and N-9 caused transient decreases in most vaginal bacteria, but N-9 use may increase *E. coli* colonization. Further evaluation of activity of these agents against sexually transmitted pathogens is required. Supported by NIH grant # AI 37830.

### DETECTION OF HUMAN IMMUNODEFICIENCY VIRUS (HIV) AND HUMAN PAPILLOMA VIRUS (HPV) IN NORMAL AND DYSPLASTIC CERVICAL TISSUE BIOPSIES BY *IN SITU* POLYMERASE CHAIN REACTION (IS-PCR).

Antonio Sison, Ashit Sarkar, Ashraf Hassanein, Joan Smith, Michael Spence, Lisa Bobroski, Farida Shaheen, Deirdre Gundy, Omar Bagasra

**Objectives:** The role of HIV and HPV in cervical dysplasia among HIV-infected women is poorly understood. Normal and dysplastic cervical biopsies from HIV-infected nonpregnant women were studied 1) to assess the frequency of detection and 2) to determine the location of HIV and HPV using the technique of IS-PCR.
**Study Design:** 37 cervical biopsies from 29 HIV-infected patients were tested for HIV and HPV using IS-PCR. Tissues were subjected to DNA amplification using conserved regions of HIV gag gene, HPV, and HLA-DQα for control. Detection was achieved with biotinylated probes.
**Results:** Median CD4 count was 282 cells/mm3. The table shows IS-PCR results for HIV and HPV in relation to the histology:

| Histology | # | # Positive HIV (%) | # Positive HPV (%) |
|---|---|---|---|
| Normal | 19 | 6 (32) | 4 (21%) |
| Mild-to-moderate dysplasia | 7 | 2 (29) | 2 (29) |
| Severe dysplasia | 8 | 3 (37) | 4 (50) |
| HPV only | 3 | 1 (33) | 0 (0) |
| HPV and dysplasia | 6 | 2 (33) | 1 (17) |

Dysplasia in the cervix did not correlate with the likelihood of detecting HIV (trend X-square, p > 0.20), but a trend was noted for HPV to be detected more often as the dysplasia worsened (p = 0.045). No correlation was found between detection of HIV/HPV and absolute CD4 (p > 0.20). For both HIV and HPV, positive signals were seen in macrophages and epithelial cells, especially showing squamous metaplasia and the superficial layers of the stratified epithelium. Overall, dysplastic cells and koilocytes on histology were not consistently positive to HIV and HPV by IS-PCR.
**Conclusions:** 1) HIV and HPV are detectable within cervical tissue by IS-PCR from HIV-infected women in one third of cases, mostly in macrophages but in epithelial cells as well, 2) there was no correlation between detection of HIV by IS-PCR to severity of dysplasia, although there was a trend for increased detection of HPV with dysplasia. These findings suggest that HIV in the cervix, unlike HPV, may actually play little or no direct pathogenic role in cervical dysplasia. (Slides will be presented.)

### CULTURE VERSUS POLYMERASE CHAIN REACTION (PCR) FOR DETECTION OF *CHLAMYDIA TRACHOMATIS* IN AN INTERMEDIATE PREVALENCE POPULATION.

KA Boggess MD, CH Livengood III MD, AP Murtha MD, and J Wrenn BS. Duke University Medical Center, Durham, NC.

**Objective:** To compare the accuracy of culture and PCR for detection of *Chlamydia trachomatis* (CT) in an intermediate prevalence population.
**Study Design:** Paired endocervical specimens first for CT culture and then for PCR were collected from 1583 unselected women at risk for CT from 12/5/95–8/31/96. Culture was performed with one blind passage, and PCR was performed in duplicate. Discrepant pairs were re-tested by PCR with the plasmid DNA primer and, in the first 600 specimens, with 2 additional target primers. To compare PCR to culture, discrepancy resolution was performed. All true + specimens were defined as any culture+, or PCR+ by ≥2 assays and all true− as culture− and PCR−, or PCR+ by ≤1 assay. Clinical data from patients with discrepant results were reviewed.

**Results:** 95/1583 (6%) were both culture and PCR+. The prevalence of CT detected by culture was 7.3%, versus 8.1% by PCR. All PCR − samples were plasmid−. There was a significant association between culture and PCR results, (P < .001, Fisher's exact test).

| | Initial results | | | Results following discrepancy resolution | | | |
|---|---|---|---|---|---|---|---|
| | + | Cult− | | PCR+ | PCR− | Cult+ | Cult− |
| PCR+ | 95 (6%) | 33 (2%) | True+ | 128 (86%) | 20 (14%) | 115 (78%) | 33 (22%) |
| PCR− | 20 (1%) | 1435 (91%) | True− | 0 | 1435 (100%) | 0 | 1435 (1005%) |

Following discrepancy resolution, the sensitivity of culture was 78%, and of PCR, 86%. The PPV and NPV of culture were 100% and 98% respectively, and the PPV and NPV of PCR were 100% and 99% respectively. Of the 20 cult+/PCR− pairs, 12 cultures were positive only on subculture. The cost/specimen for culture was $18.84 and for PCR $18.88.

**Conclusions:** A high quality culture technique remains competitive with PCR for the detection of CT in an intermediate prevalence population. The use of PCR when low amounts of chlamydia are present remains to be determined. Direct costs for PCR and culture are equivalent.

**EFFECTS OF MULTIPLE APPLICATIONS OF NONOXYNOL-9 AND BENZALKONIUM CHLORIDE ON VAGINAL TISSUES AND MICROFLORA IN A MONKEY MODEL.** DL Patton PhD, GM Ganzle Kidder, YT Cosgrove Sweeney, LK Rabe, SL Hillier PhD. Depts. of Obstetrics & Gynecology, Univ. of Washington, Seattle WA, and Magee-Womens Hospital, Univ. of Pittsburgh, Pittsburgh, PA, USA

**Objectives:** To evaluate nonoxynol-9 (N9) and benzalkonium chloride (BZK) as topical microbicides which may help control the spread of STDs. In the macaque model, we assess the effects that multiple applications of these candidate agents have on vaginal flora and lower reproductive tract tissues.

**Study Design:** Monkeys received repeated intravaginal applications of N9 (n = 4), BZK (n = 5), or N9 + BZK (n = 5). Each animal received the microbicide(s) daily for 3 to 5 days. Gross colposcopic observations and vaginal microflora assessments were performed on all animals prior to, throughout and following completion of microbicide applications. Cervical biopsies for histopathological analysis were also collected from all animals.

**Results:** At colposcopy, cervical and vaginal erythema was observed in all microbicide groups. Papillae and epithelial disruption were apparent on the cervical tissues in all groups treated with N9. Epithelial disruption of the vaginal tissues was additionally noted in the BZK only and N9 + BZK group. Histopathology of the cervical biopsies detected acute inflammatory infiltrate and an influx of plasma cells in all treatment groups, and lymphoid follicles, indicative of chronic inflammation in some specimens. Microflora assessments revealed transient decreases in anaerobic gram negative rods and peptostreptococci in all microbicide groups. N9 induced a transient increase in *Staph aureus* levels, when applied alone or in combination with BZK. Viridans levels decreased after application of BZK alone or in any combination. Lactobacilli were decreased in the BZK only treatment group. All microflora levels recovered within one week of no microbicide use.

**Conclusions:** Although N9 is currently the only microbicide approved for use as a spermicide in this country, its repeated use may be detrimental to the epithelial tissues of the female reproductive tract. BZK, which is approved for use in other countries may not only damage epithelial tissues, but also appears to reduce the population of potentially protective lactobacilli in the vagina.

Supported in part by grants P01-AI-39061 and CONRAD subproject CSA-95-161.

**EYE SPLASH IN GYNECOLOGICAL PROCEDURES.** Donnell Bradford, M.D., Andrew Helfgott, M.D., Ronald Lorimor, Ph.D.

**Objectives:** To determine the rate of face splash during gynecological procedures, and to identify the personnel and procedures with the highest risks.

**Study Design:** Opti-Shield face shields were worn during operative gynecological procedures. The masks were then collected from participants. Shields were visually examined for evidence of contamination. The face shields were subsequently immersed in hydrogen peroxide ($H_2O_2$) to confirm contamination by splash. The Gauss statistical program was used to perform Pearson's Chi-square test.

**Results:**

| | | |
|---|---|---|
| Visual splash | 157/521 | (30%) |
| $H_2O_2$ positive | 169/521 | (32%) |

| Procedures with splashes: | | | Personnel with splashes: Postgraduate year | | |
|---|---|---|---|---|---|
| Major abdominal | 73/154 | (47%) | PGY I | 17/57 | (30%) |
| Minor abdominal | 20/93 | (22%) | II | 42/156 | (27%) |
| Vaginal | 56/121 | (46%) | III | 44/112 | (39%) |
| Laparoscopic | 11/76 | (15%) | IV | 50/140 | (36%) |
| Minor | 9/77 | (12%) | Attending | 12/33 | (36%) |
| P < 0.0001 | | | Student | 4/23 | (17%) |
| | | | P < 0.0001 | | |

**Results:** Thirty two % of personnel performing gynecological procedures had face splashes as determined by $H_2O_2$ immersion. This 32% splash as determined by $H_2O_2$ had a 95% C.I. of ±4.0. PGY III, PGY IV, and attendings were exposed at the highest rate. Risk of exposure was greatest during major abdominal and vaginal surgery.

**Conclusion:** Certain gynecology procedures place surgical participants at significant risk for exposure to eye splash. This data supports the use of Opti-shield face protectors in operative gynecological procedures.

**COMPLIANCE WITH UNIVERSAL PRECAUTIONS: KNOWLEDGE AND BEHAVIOR OF RESIDENTS AND STUDENTS IN A DEPARTMENT OF OBSTETRICS AND GYNECOLOGY.** Francisco J. Garcini M.D., Ph.D., Jennifer Taylor-Burton, M.P.H., Richard Grimes, Ph.D., Andrew Helfgott, M.D., Nancy Eriksen, M.D., Jorge D. Blanco, M.D.

**Objectives:** To assess the knowledge of universal precautions (UP) for the delivery and operating rooms by residents and students, and to evaluate their use of UP.
**Study Design:** Obstetrics and Gynecology (OB/GYN) residents (n = 24) and students (n = 18) from an inner-city, teaching hospital were polled by anonymous questionnaire to assess their knowledge of the appropriate barrier equipment for certain OB/GYN procedures. To determine actual compliance with UP, 459 OB/GYN procedures were observed. We noted the use of appropriate barrier equipment for each procedure: pelvic exam (gloves), vaginal delivery, cesarean, D&C (face shields, gowns, gloves, booties). The True Epistat statistical program was used to perform linear regression analysis.
**Results:** Twenty four (100%) residents knew the appropriate barrier equipment required for each type of procedure performed. One (5%) student did not know that booties were appropriate for the surgical procedures. Rationale for lack of compliance with UP elicited by the questionnaire included: time constraints (60%), inconvenience (50%), and presumption that patient was not infected (33%).
The observed rate of compliance with UP by participants was:

| Personnel | Number of observed procedures | Gloves % | Gown % | Face Shield % | Booties % | Overall % |
|---|---|---|---|---|---|---|
| Students | 100 | 100 | 99 | 91 | 95 | 96 |
| Residents | 359 | 100 | 94 | 71 | 87 | 88 |

Individual compliance was inversely related to the years of experience (overall compliance rate of students was 96% (1st year residents 92%, 2nd year 89%, 3rd year 84%, and 4th year residents 78%), (r = −0.9918, p = 0.0009).
**Conclusions:** Knowledge regarding UP was nearly 100%, while overall observed compliance was only 89%. Compliance with UP was better among students (96%) than among residents (88%). Compliance with UP was inversely related to years of experience.

**TRANSVAGINAL SONOGRAPHY (TVS) GUIDED ASPIRATION OF PELVIC ABSCESSES (PA).** P. Corsi, M.D., S. Johnson, M.D., M.P. Diamond, M.D., S.G. McNeeley, M.D., B. Gonik, M.D. Depts. Ob/Gyn and Radiology, Wayne State University, Detroit, MI.

**Objective:** PA unresponsive to antimicrobial therapy traditionally required operative extirpation of involved tissues. A more conservative approach now advocates drainage via laparotomy, laparoscopy or transabdominal invasive radiology. Transvaginal drainage via culdotomy is usually reserved for PA "pointing" into the cul-desac. Given recent advances in TVS with biopsy guidance systems, we report our experience with this minimally invasive technique.
**Study Design:** A retrospective analysis, from 1991–1995, was carried out on 27 consecutive cases of PA (13 tuboovarian (TOA) associated with pelvic inflammatory disease; 14 postoperative (POSTOP)) undergoing TVS drainage. Demographic, clinical, laboratory and outcome data were extracted by chart review and examination of ultrasound files. Failure of the procedure was defined as persistence of symptoms and need for additional operative interventions.
**Results:** The mean patient age for the study group was 39 years. Mean duration from diagnosis to drainage was 5.6 days (TOA) and 2.0 days (POSTOP); p < 0.01. All patients received broad spectrum intravenous antibiotics from admission to resolution of acute symptoms. The mean diameter of the PA was 86 mm; from 70–750 cc of purulent material was drained with each procedure. Perceived adequacy of drainage was correlated to lack of septations within the PA. Culture results were positive in 51% of cases (79% POSTOP, 23% TOA; p < 0.05) with 1.9 organisms/positive culture. TVS drainage was successful in 25 of 27 cases (93%). Failures occurred in one TOA and one POSTOP case. There were no complications reported.
**Conclusion:** TVS guided aspiration of PA is a highly successful technique with minimal risk associated with the procedure. Given that "pointing" is not a prerequisite, this procedure might be considered earlier in the course of the disease process, when fewer septations and more adequate drainage can be anticipated. Although we speculate that this adjunctive procedure should result in an improvement of long-term outcome, follow up studies are needed to assess the occurrence of involuntary infertility, ectopic pregnancy and chronic pelvic pain.

**THE ROLE OF MATERNAL GENITAL HSV TITER AND STAGE OF DISEASE IN NEONATAL HSV TRANSMISSION.** ZA Brown[1], S Selke[3], J Zeh[3], R Ashley[2], DH Watts[1], L Corey[2]. Departments of Obstetrics and Gynecology[1], Laboratory Medicine[2] and Biostatistics[3]. University of Washington, Seattle, WA.

**Objectives:** It is uncertain what factors are involved in perinatal HSV transmission. Factors proposed include site of shedding, stage of disease, viral load, duration of fetal exposure, status of membranes, fetal scalp electrodes and type specific HSV antibody in the fetal circulation. To address this problem, we performed quantitative PCR on the genital secretions of women at the time of labor.

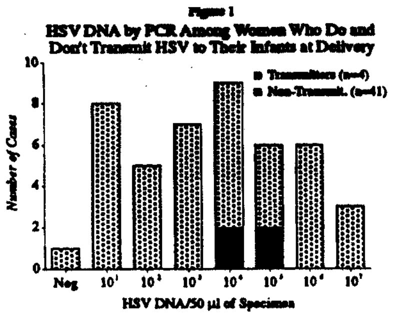

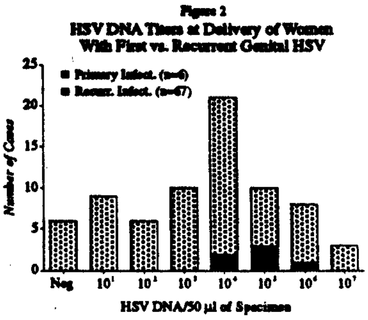

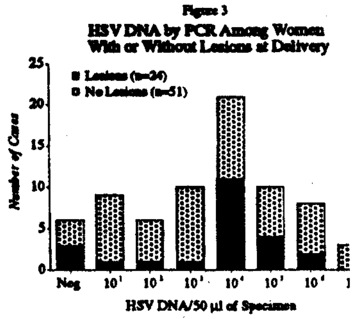

**Study Design:** Between 1989 and 1995, 17,197 women had swabs for genital HSV studies obtained at the time of labor; 108 were culture positive, 17,089 were negative. Quantitative PCR was performed on 73 of the 108 and 2 of the 17,089 women. Of the 75 women with quantitative PCR, lesions were present in 24, HSV serology was available on 73 (6 first episode and 67 reactivation) and vaginal delivery occurred in 45 (4 with neonatal transmission).
**Results:** There was no relationship between the titer of HSV DNA in genital secretions and whether neonatal HSV transmission did or did not occur (Figure 1), whether the presence of HSV in genital secretions was secondary to first episode or recurrent disease (Figure 2), or whether lesions were present or absent (Figure 3).
**Conclusions:** The quantity of HSV DNA in genital secretions does not appear to be related to the clinical stage of maternal disease, the presence or absence of clinical lesions and does not appear to be a major determinant for HSV neonatal transmission.

### INFLUENCE OF THE NORMAL MENSTRUAL CYCLE AND COMBINATION ORAL CONTRACEPTIVES ON VAGINAL EPITHELIUM AND FLORA.

DL Patton PhD, SS Thwin MS, KJ Agnew BS, DA Eschenbach MD. University of Washington, Seattle, WA.

**Introduction:** The effect of hormone levels on vaginal epithelial thickness may influence the transmission of STDs.
**Objective:** Examine vaginal tissue and flora during three phases of the menstrual cycle, and before initiation and after two months of combination oral contraceptive (OC) use.
**Study Design:** We examined vaginal tissue in two groups of women. Vaginal biopsies and cultures were obtained during three phases (d1–5, d7–12, d19–24) of the normal menstrual cycle (n = 52) or before initiation and after two months of combination OC (35 µg of ethinyl estradiol and 1 mg of norethindrone) use (n = 27). Histologic and microbiologic assessments were made by observers blinded to clinical data.
**Results:** In the normal menstrual cycle group, the mean number of epithelial cell layers underwent a small but significant decrease from 29.7 ± 0.8 on days 1–5 to 28.9 ± 0.7 on days 7–12 and 26.9 ± 0.7 on days 19–24 of the menstrual cycle ($p = 0.03$). Non-ovulating women had a reduced mean epithelial cell layer count on days 7–12 (23.7 ± 1.3) compared to ovulating women (29.8 ± .9, $p = 0.003$).
The mean number of epithelial cell layers on days 19–24 prior to (29.6 ± 1.1) and after two months of combination OC use (29.7 ± 1.1) was similar ($p = 0.9$). The number of polymorphonuclear leukocytes, inflammatory foci, lymphoid follicles, plasma cells and macrophages was minimal and similar in all groups studied.
Among women with any vaginal *Lactobacillus*, the proportion with moderate to heavy growth of other vaginal bacterial species (>$10^5$ bacteria/ml) decreased from 61% (25/41) at 1–5 days to 31% (15/49) at 7–12 days and 46% (23/50) at 19–24 days of the cycle ($p = 0.02$). No such change occurred in women with no vaginal *Lactobacillus* or after OC use:
**Conclusion:** Small but significant differences in vaginal epithelial thickness and flora were seen during the menstrual cycle. Oral contraceptive use did not appear to influence vaginal epithelial thickness or flora.

Supported by R01 HD 33203, NICHD, Contraceptive Development Branch.

### A COMMENSAL RELATIONSHIP BETWEEN *PREVOTELLA BIVIA* AND *PEPTOSTREPTOCOCCUS ANAEROBIUS* INVOLVES AMINO ACIDS: POTENTIAL SIGNIFICANCE FOR BACTERIAL VAGINOSIS.

Vivien Pybus, PhD and Andrew B. Onderdonk, PhD.

**Objectives:** Bacterial vaginosis (BV) has a complex microbiology which includes anaerobes such as *Prevotella bivia* and *Peptostreptococcus anaerobius,* and the facultative organisms *Gardnerella vaginalis.* An understanding of interactions between BV-associated organisms can contribute to the knowledge on both the pathogenesis and maintenance of this syndrome. The objective of the current study was to describe any symbiosis between *P. bivia* and *P. anaerobius.*
**Study Design & Results:** We observed that *P. anaerobius* was unable to grow in pure culture in vaginal defined medium (VDM), anaerobically in both batch and continuous culture. However, under the same conditions, when *P. bivia* was simultaneously present, *P. anaerobius* growth was supported. In quadruplicate experiments the maximum detectable concentration of *P. anaerobius* increased from a mean of $\log_{10}$ 8.57 cfu/mL in peptone-supplemented VDM to $\log_{10}$ 8.94 cfu/mL ($P = 0.014$) when grown in the same medium conditioned by prior growth of *P. bivia.* Collectively, these results suggest that *P. bivia* stimulates the growth of *P. anaerobius,* and that a commensal symbiosis may exist between these organisms.

To chemically define this relationship, culture supernatants from *P. bivia* growth were analyzed for amino acid content relative to control (uninoculated) media. The following amino acids produced by *P. bivia* (in the decreasing concentration range indicated) were subsequently shown to be utilized by *P. anaerobius* during growth in *P. bivia*-conditioned supernatants: alanine (1.5 mM), glycine, leucine, glutamate, phenylalanine, tyrosine, valine, aspartate, methionine, lysine and proline (0.15 mM).
**Conclusions:** Amino acid flow from *P. bivia* to *P. anaerobius* is proposed as a mechanism to support a commensal relationship between these organisms. We have previously found *P. bivia* growth stimulatory for *G. vaginalis*[1], and the concentration of *Prevotella*

spp.[2] to be an important predictor of the health of the vagina. Potentially, these results underscore the significance of elevated, vaginal carriage of *Prevotella* spp.

**References:**

1 Pybus, V. and A.B. Onderdonk (1997). *Journal of Infectious Diseases* **175**:406–415.
2 Lee, M.-L.T., Ross, R.A., Delaney, M.L. and A.B. Onderdonk (1994). *Microbial Ecology in Health and Disease* **7**:235–240.

*Best Abstract Award*

**HEPATITIS C VIRUS INFECTION IN PREGNANCY: IS MATERNAL VIRAL BURDEN RELATED TO VERTICAL TRANSMISSION?** NS Silverman, MD, FN Jaffee, MD, RL Hodinka, PhD. Department of Obstetrics and Gynecology, Division of Maternal-Fetal Medicine, Thomas Jefferson Medical College of Thomas Jefferson University Hospital, and Departments of Pediatrics and Pathology, Children's Hospital of Philadelphia, Philadelphia, PA.

**Objective:** We performed quantitative evaluation of serum titers of hepatitis C virus (HCV) in a cohort of substance-addicted, HCV-RNA-positive pregnant women to test the hypothesis that the level of maternal viremia near term was related to the risk of vertical HCV transmission.

**Study Design:** All 23 HCV-antibody-positive women identified prospectively through routine screening over a 15-month period gave consent for additional blood to be drawn from them, at 32–36 weeks, and from their newborns. These study samples were analyzed for the presence of HCV-RNA-specific sequences via qualitative PCR, with positive samples then quantitatively tested via a branched DNA (bDNA) assay with a lower level of sensitivity of 0.2 HCV-RNA MEq/ml.

**Results:** Of the 23 anti-HCV-positive women in this study, 18 (78%) were HCV-RNA-positive, with paired maternal-neonatal samples available for 17 of these 18 pregnancies (one term fetal demise). Three antibody positive women were also HIV-infected, with 2 among the 18 (11.1%) in the HCV-RNA-positive group. One of the 17 infants (5.9%) born to RNA-positive mothers had HCV-RNA detected in its serum; this mother was also HIV-infected. Women who were RNA-positive had a higher mean SGOT than those who were antibody-positive but RNA-negative (28.2 ± 13.2 vs 12.8 ± 2.3, $p = 0.02$); their median bDNA titer was 2.76 MEq/ml (range 0.2–61.8). Median bDNA titers for women with normal and abnormal SGOT (cutoff 30 IU/l) were not significantly different (1.2 vs 3.4 MEq/ml; $p = 0.88$ by Mann-Whitney). The 2 RNA-positive HIV-infected women had low bDNA titers (0.46 and 0.28 MEq/ml), with the HCV-transmitting mother's value the lower of the two.

**Conclusions:** The risk of vertical HCV transmission in this high-risk cohort was 6%, and was related more to maternal HIV status than HCV viral titer by quantitative assay. There was no relation seen between maternal HCV titer and liver function test abnormalities.

**AN OFFICE-BASED INTERVENTION TO IMPROVE VACCINATION OF PATIENTS AT RISK FOR SEXUAL TRANSMISSION OF HEPATITIS B VIRUS INFECTION.** Harold S. Margolis, MD, H. Hunter Handsfield, MD, R. Jake Jacobs, MPA, Joseph E. Gangi, BS.

**Objectives:** Assess the frequency with which patients at risk of sexually transmitted hepatitis B virus (HBV) receive counseling and vaccination from private practice physicians, and assess the impact of raising physician awareness and offering intervention tools.

**Study Design:** Materials were provided to assist in both retrospective identification of patients at risk of sexually acquired HBV infection, and counseling concerning HBV infection prevention. The program's effectiveness was assessed in a study conducted in practices across the United States. Physicians were randomized to either an intervention or current practices group. The intervention group used letters, educational materials and follow-up telephone calls to encourage patients to make an appointment to discuss HBV prevention and vaccination. Physicians in the current practices group received no identification, contact or counseling materials.

**Results:** Forty-nine practitioners (13 family physicians, 36 OB/GYNs) provided baseline data on 457 patients at risk of sexually transmitted HBV. Twenty-eight physicians provided follow-up information for 226 patients. At baseline, only 6.5% of patients were counseled regarding risks of HBV infection, 2.4% started a vaccination series, and 0.7% had completed vaccination. Counseling was least likely to occur in OB/GYN practices, among uninsured patients, and among patients without a risk factor other than a recent sexually transmitted disease. Following the six-month study period, 44% in the intervention versus 15% in the current practices group received HBV counseling ($p < 0.01$). Patients in the intervention group were also more likely to begin (12.6% versus 0.9%, $p < 0.01$) and complete (4.8% versus 0.0%, $p = 0.03$) a vaccination series.

**Conclusions:** Prospective use of this intervention for identification, counseling, and vaccination of high-risk women should be evaluated.

**EVALUATION OF U.S. PERINATAL HEPATITIS B VIRUS PREVENTION ACTIVITIES.** Frank J. Mahoney, MD, and Nicole M. Smith, MPH, MPP, Hepatitis Branch, CDC

**Objectives:** To estimate progress toward the national disease reduction goal and objectives for preventing perinatal transmission of the hepatitis B virus.

**Study Design:** Surveys of hepatitis coordinators from federally-funded projects were conducted in 1994–1996. Medical record reviews and hospital policy surveys in five states provided additional information about maternal hepatitis B surface antigen (HBsAg) screening rates and vaccination practices for infants born to HBsAg+ mothers.

**Results:** Coordinators reported that estimated maternal screening rates vary from <50% to >90%. However, rates in the five states ranged from 88% to 96%. Several risk factors for lack of maternal HBsAg screening were identified, including absence of hospital policies for maternal HBsAg testing, absence of prenatal care and public assistance payment for delivery. During the 1993 to 1995 time period, 7691–8258 annual births to HBsAg+ women were identified, compared to the 20,000 births estimated to occur. States identifying ≥90% of the expected number of births to infected mothers include those that have computerized tracking systems, hospital reporting systems, and universal reporting of maternal HBsAg status on newborn screening cards/birth certificates. Of the identified infants, 88–93% received hepatitis B vaccine and HBIG at birth, and 61–71% completed the vaccine series by 6–8 months of age. Between 15–22% of identified infants had post-vaccination serologic testing and each year only 3% were HBsAg-positive.

**Conclusions:** Activities to prevent perinatal HBV transmission have been well-integrated into routine prenatal care. However, universal HBsAg screening of pregnant women has not been achieved and comprehensive case management of infants born to HBsAg-positive women has not occurred. Improved efforts are necessary if the number of perinatal HBV infections is to be reduced 80% by the year 2000.

### THE ERADICATION OF HUMAN PAPILLOMA VIRUS IN WOMEN WITH CONDYLOMA ACUMINATA.

Stanley A. Gall, M.D.; Zhemin Lei, Ph.D.; C.V. Rao, Ph.D.

**Objectives:** To determine whether a prolonged course of alpha interferon could eliminate human papilloma virus.
**Study Design:** A pilot study of ten women were treated with lymphoid interferon via systemic subcutaneous infection with a dose of 2.5 million units three times weekly, self administered for six months. Vulvar biopsy of the wart and subsequently the wart area were done at the initiation of the study and for six consecutive months. Photographs were taken of the vulva on each visit. The biopsies were examined by autoradiography and by polymerase chain reaction. (PCR) The patients clinical course was followed. Follow up time has been greater than five years.
**Results:** All ten patients in the pilot study cleared their condyloma acuminata during the course of therapy. In the follow up five year period, five patients had recurrence of their condyloma acuminata but five patients have not had recurrence. The autoradiography results showed a progressive diminution in the presence of HPV with each subsequent biopsy in the five patients whose condyloma did not recur while continuing positive results were seen in patients whose condyloma subsequently recurred. The PCR results showed the absence of HPV on the final biopsies of those women who condyloma has not recurred in five years of follow up.
**Conclusions:** The therapy of condyloma acuminata with six months of interferon in a protocol similar to the Hepatitis C protocols, caused the elimination of HPV in five of ten women. The same five women who became PCR negative have not experienced a recurrence of condyloma acuminata after a five year follow up.

### POSTER SESSIONS

### WOMEN'S BELIEFS ABOUT STD.

Edward R. Newton M.D., Mark Funk, M.D., Rochelle Shain, Ph.D., Jeanna Piper, M.D., Sandra Perdue, DPH

**Objective:** We sought to measure women's knowledge about STD.
**Study Design:** In a six week period, 628 of 638 (98%) unselected, consecutive women attending general gynecology clinics at the Robert B. Green Outpatient Center filled out a short questionnaire which surveyed their demographics, STD history, and their STD knowledge (17 multichoice questions). These questions measured the woman's assessment of partner risk, symptoms of STD, consequences of STD, STD acquisition, and response to treatment.
**Results:** The characteristics of the population were: Black (34%), Hispanic (52%), Anglo (8%), other race (6%), <21 y/o (31%), <12$^{th}$ grade education (49%), IV drug use (2.8%), H/O 1 STD (24%), H/O > 1 STD (32%), nulligravida (34%). The women gave wrong answers in 34% (±16%) of questions. The common errors (% wrong) concerned: symptoms in women (77%), natural history of untreated STD (98%), acquisition from oral sex (32%), and infertility from STD (53%), the use of drugs or alcohol with problems using condoms (74%). Twenty percent of women did not consider AIDS a STD.
**Conclusions:** Minority women of lower socioeconomic status have fair knowledge concerning STD. Educational efforts might focus on symptoms (or lack of) in women, long term consequences, barriers to condom use, and mechanisms of acquisition.

### THE INCREASED INCIDENCE OF GYNECOLOGIC DISEASES IN HIV INFECTED WOMEN: THE ROLE OF HIV INFECTION VERSUS OTHER RISK FACTORS.

Riley L*, Jarek C, Steger K, Craven D. Mass General Hospital* and Boston Medical Center, Boston, MA.

**Objectives:**
1. To compare rates of specific gynecologic abnormalities among a group of HIV-infected women (cases) versus HIV-negative high risk women (controls).
2. To assess these rates by other risk factors, such as injection drug use (IDU) and numbers of lifetime sexual partners.

**Study Design:** We prospectively performed gynecologic examinations, collected routine cultures and relevant medical data on 232 cases and 20 controls from November, 1992–December, 1996.
**Results:** Cases and controls were similar in terms of age (35.2 yrs. vs 35.1 yrs. respectively). Cases were 32% white, 39% black, 13% Latino, 11% Haitian; controls were 55% white, 35% black, and 10% Latina. IDU was reported by 53% of cases and 70% of controls. Twenty-eight percent of cases and 25% of controls reported having >21 lifetime sexual partners (NS). Gynecologic abnormalities either reported in the patient's history or diagnosed on exam:

| GYN disease | HIV-infected n = 232 | HIV-negative n = 20 |
|---|---|---|
| Gonorrhea | 86 (37%) | 8 (40%) |
| Chlamydia | 50 (22%) | 5 (50%) |
| PID | 47 (20%) | 6 (30%) |
| HPV | 42 (18%) | 5 (25%) |
| HSV | 44 (19%) | 1 (5%) |
| Dysplasia* | 73 (31%) | 0 |
| Amenorrhea | 70 (30%) | 6 (30%) |

*Dysplasia was the only abnormality which was significantly different between the groups ($p = 0.0013$). Of those women reporting any STD, cases had a 3.5 fold increase in recurrent Chlamydia and a 2.4 fold increase in recurrent gonorrhea over controls.

**Conclusions:** Sexually transmitted diseases (STD) were common in our population of HIV-infected women and high risk HIV seronegative controls. The cases reported more recurrences of GC and Chlamydia than controls. The rate of dysplasia was significantly increased in HIV-infected women. Amenorrhea was predominantly associated with active dry users in both groups. The high rate of gynecologic abnormalities among HIV-infected women may be due, in part, to other lifestyle risk factors.

**TOUGH TO TREAT TRICHOMONIASIS: RESULTS WITH PAROMOMYCIN CREAM.** P Nyirjesy, MD; JD Sobel, MD; MV Weitz, MSN; DJ Leaman, BSN; SP Gelone PharmD. Temple University School of Medicine, Philadelphia, PA and Wayne State University School of Medicine, Detroit, MI.

**Objective:** To assess the efficacy of 6.25% paromomycin cream in the treatment of women referred with vaginal trichomonal infections where metronidazole resistance or allergy was present.
**Study Design:** The records of 6 patients who were treated with paromomycin cream were reviewed. Trichomoniasis was diagnosed by wet mount with culture confirmation as needed. Patients used a 4g applicator of cream (250 mg of paromomycin) nightly intravaginally for 14 days. Results immediately and 1 month after treatment were reviewed.
**Results:** The median age was 43 (range 29–49). Three patients were nulliparous. The median symptom duration was 7.5 years (range 0.5–14). Three women were allergic to metronidazole. Three had infections which were resistant to high dose metronidazole. All 6 patients had negative saline preparations after treatment, but 2 had positive cultures. A third patient's infection relapsed two weeks after treatment. Of these failures, one was later cured with a 3-week course of paromomycin cream. Two patients, both at the same clinical center, developed vaginal ulcerations during treatment which resolved spontaneously.
**Conclusions:** Paromomycin cream cured ⅔ of trichomonal infections where metronidazole resistance or allergy was encountered. Adverse effects may have been a result of the local formulation.

**4% CROMOLYN CREAM FOR VULVAR VESTIBULITIS: RESULTS OF A PILOT STUDY.** Paul Nyirjesy, M.D.; Maria J. Small, M.D.; M. Velma Weitz, M.S.N.; Paula K. Braverman, M.D.; Steven P. Gelone, Pharm.D. Temple University School of Medicine, Phildelphia, PA.

**Objective:** To assess the efficacy of 4% cromolyn cream in the treatment of women with recalcitrant vulvar vestibulitis.
**Study Design:** The records of 7 patients who were treated with 4% cromolyn cream for vulvar vestibulitis were reviewed. Vulvar vestibulitis was diagnosed by standard criteria. Patients received 6 weeks of topical cream, applied tid to the vestibule. Analysis of their symptoms at the end of treatment was performed.
**Results:** The median age was 28 years (range 23–46). Six patients were nulliparous. The median duration of symptoms was 2.5 years (range 0.7–6). Before treatment, mean symptom severity score was $2.7 \pm 0.5$ for irritation, $1.7 \pm 1.3$ for burning, $1.0 \pm 0.8$ for itching, and $2.6 \pm 0.6$ for dyspareunia, where a 0–3 none to severe symptom scale was used. 4 patients had pre-existing vulvovaginal candidiasis with persistence of symptoms despite effective antifungal treatment. All patients had failed therapy with topical 0.1% triamcinolone ointment; 6 had failed a course of amitriptyline. After treatment, all patients had no symptoms with daily activity. Dyspareunia decreased to a score of $1.2 \pm 0.7$ in the 5 patients who were sexually active. 6 patients felt they were profoundly better; 1 noted moderate improvement.
**Conclusions:** 4% cromolyn cream may be an effective treatment for vulvar vestibulitis. Further controlled studies will be necessary.

**CUTANEOUS ANERGY IN PREGNANT AND NON-PREGNANT WOMEN WITH HUMAN IMMUNODEFICIENCY VIRUS.** N.L. Eriksen, A.W. Helfgott. UTHSC—Houston.

**Objective:** To determine the prevalence of cutaneous anergy in pregnant and non pregnant women with HIV infection.
**Study Design:** The medical records of 159 women seropositive for human immunodeficiency virus were reviewed. Demographic characteristics and tuberculin skin test results were abstracted from the chart. Tuberculin skin testing was performed by the Mantoux method (5 TU of PPD) injected intradermally. Anergy testing was performed using any two of the three following antigens; tetanus toxoid, mumps or candida skin test antigen. A positive tuberculin test was defined as induration of 5 mm or more and a positive anergy test was defined as any amount of induration over the anergy skin test area. A CD4+T lymphocyte count was obtained at the time of skin testing. Continuous variables were analyzed using the Mann Whitney-U test. Categorical data were analyzed with the Chi-Square or Fishers' Exact test as appropriate. A two-tailed p value < 0.05 was considered significant.
**Results:** Tuberculin skin test results are shown below:

| | Non pregnant (n = 102) | Pregnant (n = 57) |
|---|---|---|
| Positive | 13 (12.7%) | 5 (8.7%) |
| Negative | 62 (60.7%) | 37 (64.9%) |
| Anergic | 27 (26.6%) | 15 (26.4%) |

There was no significant difference in the prevalence of positive, negative or anergic skin test results between groups. The CD4+T lymphocyte count (mean ± standard deviation) in patients with anergic results was similar between pregnant ($375/mm^3$) and non-pregnant ($358/mm^3$) women ($p = 0.64$).
**Conclusion:** The prevalence of cutaneous anergy is similar in pregnant and nonpregnant women with human immunodeficiency virus.

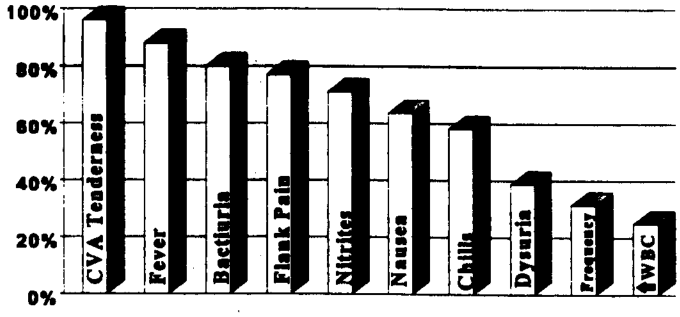

The CD4+T lymphocyte count was not statistically different in pregnant versus non-pregnant women with anergy.

### IMPROVED RELIABILITY OF DIAGNOSIS OF BACTERIAL VAGINOSIS USING AN OBJECTIVE DEVICE FOR DETECTION OF ELEVATED VAGINAL PH AND TRIMETHYLAMINE.

SL Hillier, J Schwebke, J Sobel. J McGregor, RL Sweet. University of Pittsburgh, PA, University of Alabama at Birmingham, Birmingham, AL, Wayne State University, Detroit, MI, University of Colorado, Denver, CO.

**Objective:** To evaluate interobserver reproducibility for performance of vaginal pH and amine odor determinations, and to assess the utility of an objective colorimetric test for vaginal pH and trimethylamine.

**Study Design:** Interobserver reproducibility of pH was assessed among 13 individuals who used pH paper to test solutions of pH 4.0–5.0. Individuals were also asked to evaluate stock solutions containing 0.05–50.0 mM trimethylamine for amine odor in a blinded manner. Observers evaluated a pH/amine detection card (Fem Exam™ TestCard, Litmus Concepts, Santa Clara, CA) in the same manner. Utility of the pH/amine detection card was compared to traditional pH/amine measurements in a multicenter study of 607 women.

**Results:** Using ColorpHast pH test strips, 23% of the observers incorrectly identified the pH 4.5 solution as having a pH of 4.7, while 100% of the participants correctly interpreted the pH 4.5 as negative using the Fem Exam™ TestCard. For detection of amine odor, only 66% of observers detected 5 mm of trimethylamine, while 100% of observers accurately detected trimethylamine using the colorimetric card test.

**Conclusions:** Traditional pH and amine testing of vaginal fluid does not have optimal sensitivity or specificity. An objective rapid colorimetric test for pH and trimethylamine provides better specificity for vaginal pH and improved sensitivity for trimethylamine. These two test elements provide a rapid presumptive test for bedside diagnosis of BV. The clinical study demonstrated that 163 (89.6%) of 182 women positive for BV by both Gram stain and Amsel criteria were positive for the pH/amine card test, compared to 13 (3%) of 425 women negative for BV by Gram stain and Amsel criteria.

### ACUTE PYELONEPHRITIS: DIAGNOSTIC CRITERIA AND CHARACTERISTICS.

J.M. Piper, M.D., S.K. Zavala, M.D., S.C. Fox, E.M-J. Xenakis, M.D., O. Langer, M.D.

**Objective:** To define the diagnostic criteria for acute pyelonephritis in pregnancy, and examine the clinical characteristics.

**Study Design:** Consecutive pregnant women admitted to the hospital with a diagnosis of acute pyelonephritis from June 1993 to May 1996 were analyzed. Demographic data, previous genitourinary and medical history, presenting signs and symptoms, admission examination and laboratory findings, antibiotic usage and hospital course were evaluated. Only cases with a positive urine culture and fever at home or on admission were included.

**Results:** 126 women were admitted with acute pyelonephritis in pregnancy over the 3-year study period (incidence 1.1%). Infection was most common in the mid-trimester [52% vs. 19% (1st), 29% (3rd)]. 44% had not yet sought prenatal care. This cohort was young (35% <20 yrs, 93% <30yrs) and evenly split between multiparas (55%) and nulliparas (45%). 37% had a prior UTI this pregnancy, 57% of which were incompletely treated.

**Conclusion:** CVA tenderness/Flank pain and urine bacteria/nitrites in the presence of fever are diagnostic for pyelonephritis in pregnancy. Lower urinary symptoms, in contrast, are not useful for diagnosis. Inadequate treatment of lower tract infections is a significant contributor to development of pyelonephritis.

### DETECTION OF CHLAMYDIA TRACHOMATIS ANTIGENIC MATERIAL IN HUMAN OVARIAN, PROSTATIC AND SEMEN SAMPLES.

Toth M, Patton DL, Campbell LA. Dept of Obstetrics & Gynecology, Cornell University Medical College, New York, NY, and Depts of Obstetrics & Gynecology and Pathobiology, University of Washington, Seattle, WA, USA

**Objectives:** It has been hypothesized that a delayed hypersensitivity reaction in the host to chlamydial antigenic material may lead to irreversible scarring of the reproductive tract. We used in situ hybridization (ISH) and immunoperoxidase staining (IP) on human ovarian, prostatic and semen samples to identify chlamydial antigenic material.

**Study Design:** Detection of chlamydia was achieved by two methods, IP and ISH. Tissues assessed by IP stain were labeled with one of two antibodies. The monoclonal antibody KK12 recognizes a *C trachomatis* species-specific MOMP determinant, and a separate monoclonal antibody, CF2 which recognizes genus-specific MOMP determinant and a separate monoclonal antibody, CF2 which recognizes genus-specific LPS. ISH detects chlamydial DNA. Ovarian biopsy specimens from 19 culture negative women with pelvic adhesions and/or tubal factor infertility were analyzed by both methods. Samples of prostates from 10 men undergoing prostatectomy for benign hypertrophy were analyzed by IP stains. In addition, semen specimens from culture negative sexual partners of eight women with PID and/or bacterial vaginosis were tested by IP methods only.

**Results:** Seven of the 19 ovarian specimens tested positive for chlamydia antigen or DNA (36.8%). Both chlamydial LPS and MOMP were readily identifiable in the prostate tissues. Of the 10 hypertrophic prostates examined, 4 (40%) were positive by any method. Of the nine samples of semen tested by IP, only one (11%) was positive.

**Conclusions:** 1. There is a high prevalence of antigenic material in the ovaries of chronically infected, though culture negative women. 2. Chlamydia antigens may have an etiologic role in benign prostate hypertrophy. 3. Antigenic material may be transmitted via the semen of culture negative men.

**A RANDOMIZED CONTROLLED TRIAL OF ORAL LEVOFLOXACIN IN THE TREATMENT OF EXPERIMENTAL POLYMICROBIAL PUERPERAL INFECTION IN THE RABBIT.** Leonid Reznikov, MD, Ph.D, Joan Eskens, MS, Robert McDuffie, MD, Ronald Gibbs, MD, University of Colorado and Kaiser Permanente, Denver, CO

**Objectives:** To evaluate the efficacy of oral levofloxacin in the treatment of experimental polymicrobial puerperal infection in a rabbit model.
**Study Design:** Timed pregnant rabbits on day 29–30 of a 31 day gestation were anesthetized, and an endoscope was placed into the vagina and advanced to the cervices, both of which were inoculated with $10^{5-6}$ c.f.u. of each of *E. coli,* group B streptococcus (GBS), and *Peptostreptococcus* sp. Labor was induced with intramuscular oxytocin 16 hrs. later if it had not occurred spontaneously. Does were then observed every 3 hours for fever (>104°F.). Treatment was begun after development of fever. Animals were randomly assigned in a blinded, placebo-controlled manner to oral levofloxacin (10 mg/kg/day mixed in Nutri-Cal) or placebo (Nutri-Cal) and treated b.i.d. for 4–5 days. Necropsy was performed 4–6 hrs after the last dose. Cultures were taken from uterine horns, peritoneum, and blood. Levofloxacin concentrations were determined from blood at necropsy. Outcomes were: clinical cure of fever, eradication of microbes and presence of uterine abscesses at necropsy. Prior to the study, a sample size was calculated. Data were analyzed by Fisher's exact test. A p value of <0.05 was considered significant.
**Results:**

| Outcome | Levofloxacin | Placebo | p Value |
|---|---|---|---|
| Clinical cure | 9/11 (82%) | 4/12 (33%) | 0.027 |
| Eradication of *E. coli* | 10/11 (91%) | 5/12 (42%) | 0.022 |
| Eradication of GBS | 5/11 (45%) | 4/12 (33%) | 0.68 |
| Uterine abscess | 0/11 | 4/12 (33%) | 0.09 |

No blood cultures were positive in any animal. Levofloxacin was detected in all treated animals, but at low levels (<1 mcg/ml).
**Conclusion:** Treatment of experimental puerperal infection with oral levofloxacin resulted in significantly more clinical cures and eradication of *E. coli* compared to placebo. Sponsored by a grant from Ortho-McNeil Pharmaceutical.

**PERINATAL OUTCOME AND INFECTIOUS MORBIDITY IN PREGNANT WOMEN WHO HAVE AND HAVE NOT RECEIVED PRENATAL CARE.** Antonio Sison, Kristine Maxwell, David Adler, Edward Gracley, Michael Spence

**Objectives:** Specific risks to which pregnant women who deliver without prenatal care are subject have not been clearly identified. Our goals are 1) to assess maternal and neonatal outcome and 2) to identify risk factors in pregnancies complicated by absence of prenatal care when compared to an age-matched cohort who received prenatal care in an urban inner city population.

**Study Design:** Outcome measures from pregnant women who presented to Labor and Delivery with no prenatal care (NPC group) were collected from 1993 through 1996. Data on maternal history and outcome were recorded and then compared with an age-matched, parity-matched control group who received prenatal care (PNC group) in an inner city resident-run clinic. Drug abuse referred to crack-cocaine but also included heroine, cannabis, pencyclidine, and ethanol.
**Results:**

| | NPC group (n = 234) | PNC group (n = 95) |
|---|---|---|
| History of drug abuse | 165 (71%) | 25 (26%), p<0.001 |
| History of STD's | 102 (44%) | 45 (47%) |
| Gestational age at delivery | 34.8 weeks | 38.5 weeks |
| Preterm deliveries (≤37 weeks) | 138 (59%) | 16 (17%), p<0.001 |
| Type of delivery: | | |
| Vaginal | 158 (87%) | 80 (85%) |
| Operative vaginal | 9 (5%) | 3 (3%) |
| Cesarean | 15 (8%) | 12 (12%) |
| Hospital length of stay | 2.05 days | 1.85 days |
| Positive urine drug screen | 126 (54%) | N/A |
| Reactive test to syphilis | 40 (17%) | 4 (4%), p<0.01 |
| Mean birth weight | 2491 grams | 3158 grams |
| Apgars scores (≤5 at 1 min or ≤7 at 5 mins) | 79 (34%) | 16 (17%), p<0.01 |

**Conclusions:** 1) When compared to the PNC group, the NPC group were at risk for having a maternal history of drug abuse, earlier gestational age at delivery, higher preterm delivery rates, reactivity to the syphilis test, and lower apgar scores, 2) lack or absence of prenatal care did not appear to affect the cesarean section rate or the maternal hospital length of stay, and 3) more than half of the NPC patients presented to Labor and Delivery with a positive urine drug screen, the majority of which was for cocaine metabolites.

**THE EFFECTS OF ESCHERICHIA COLI HEAT-STABLE (STa) TOXIN OVER HUMAN MYOMETRIUM CONTRACTILITY "IN VITRO".** Carrera-Leal, B Leal de Carrera, A Pierdant PG, Deleon DF.

**Objective:** The purpose of the study was to assess the effects of Escherichia coli heat stable (STa) toxin on isolated human myometrium response to oxytocin. Method: 116 muscle strips were obtained from the lower uterine segment of 42 women undergoing Cesarean section. Uterine response to oxytocin was recorded before and after the incubation with this toxin. A $pD_2$ for strips with and without spontaneous activity in response to oxytocin before and after the incubation with STa toxin or vehicle was calculated. A paired T test was used for comparison.
**Results:** Muscle strips with and without spontaneous activity responded to cumulative doses of oxytocin before and after the incubation with STa toxin or vehicle. No differences in contractility were noted in between the groups ($p < 0.05$). Furthermore, this toxin was not able to induce uterine contractility when tested alone.
**Conclusions:** The inability of this toxin to affect myometrium response to oxytocin may be due to the lack of amnion cells or the absence of STa receptors in this tissue.

**VIRAL RECOVERY AND ENDOMETRITIS RATES IN WOMEN UNDERGOING CESAREAN FOR GENITAL HERPES LESIONS.** L.M. Hollier, D. Dawson, L.L. Scott, and G.D. Wendel, Dept. Ob/Gyn, Univ. TX Southwestern Med. Ctr., Dallas, TX

**Objectives:** To determine the rate of herpes virus recovery from cultures taken at delivery and the frequency of puerperal endometritis in women delivered by cesarean section for symptomatic genital herpes.
**Study Design:** Retrospective chart review of women undergoing cesarean delivery for genital lesions between 1/1/88 and 12/31/95 at Parkland Memorial Hospital. Cultures of the cervix, active lesion site, and usual lesion site were taken prior to cesarean delivery. Cultures were performed by the Virology Lab at the University of Texas Southwestern Medical Center. Women taking acyclovir at delivery were excluded.
**Results:** Study criteria were met by 197 women.

| | 1st Episode (N = 84) | Recurrent (N = 113) |
|---|---|---|
| Positive delivery culture | 26/60 (43%) | 27/7 (40%) |
| Lesion duration | | |
| Positive culture | 4.2 days | 3.0 days |
| Negative culture | 5.4 days | 4.5 days |
| Endometritis | 19/84 (23%) | 20/113 (18%) |

Fourteen women were having prodromal symptoms alone at delivery. None had a positive culture. No infants in this cohort developed neonatal herpes (attack rate 0.00 (95% CI, 0.00–1.86). Only one infant had a positive culture; he exhibited no clinical disease.
**Conclusions:** Viable virus was recovered from a higher percentage of our patients with recurrent disease than is generally reported. Endometritis complicates the postpartum course of 20% of women undergoing cesarean for symptomatic genital herpes.

**MATERNAL SERUM GRANULOCYTE COLONY STIMULATING FACTOR (G-CSF) CONCENTRATIONS IN PRETERM PREMATURE RUPTURE OF MEMBRANES (PPROM) AND CHORIOAMNIONITIS.** AP Murtha MD, KA Boggess MD, and PC Greig MD. Duke University Medical Center, Department of OB/Gyn, Durham, NC.

**Objective:** Preterm birth has been linked with intrauterine infection and inflammation. Serum and amniotic fluid markers of inflammation have been associated with clinical and subclinical chorioamnionitis and preterm delivery. This study was designed to determine if maternal serum G-CSF levels are elevated in patients with PPROM and intrauterine infection.
**Study Design:** Maternal serum samples were obtained from 21 patients with PPROM at 22–34 weeks gestation on the day of delivery. Maternal serum G-CSF concentrations were determined using a specific ELISA kit (R&D Systems, Inc). Clinical and histologic chorioamnionitis were defined by standard criteria. Statistical analysis was by Mann Whitney U test.
**Results:** Of the 21 PPROM patients, 13 (62%) had clinical evidence of infection on the day of delivery. Maternal serum G-CSF concentrations were significantly higher when clinical chorioamnionitis was present compared to those without clinical chorioamnionitis (146 vs 56 pg/ml, p = .026). Placental pathology was available in 20 patients. Histologic chorioamnionitis was present in 19 patients, of these, 8 (42%) were without clinical evidence of infection. Serum G-CSF concentrations were significantly higher in PPROM patients with clinical and histologic chorioamnionitis when compared to those with histologic chorioamnionitis alone (126 vs 36 pg/ml, p = .035).
**Conclusions:** Intrauterine infection causes a local inflammatory response resulting in the production of various mediators, including G-CSF. The systemic release of G-CSF is more pronounced in PPROM patients with clinical chorioamnionitis, suggesting that G-CSF may not be an early mediator in subclinical infection in patients with PPROM.

**A COST EFFECTIVENESS EVALUATION OF INPATIENT PELVIC INFLAMMATORY DISEASE TREATMENT.** Mazzoni MM, Ransom SB, Hendrix SL, McNeeley SG. Division of Gynecology, Wayne State University/Hutzel Hospital, Detroit, Michigan.

**Objective:** To determine the most cost effective antibiotic regimen for treating inpatient pelvic inflammatory disease in an urban, university-affiliated, teaching hospital.
**Study Design:** A retrospective evaluation for the treatment of inpatient pelvic inflammatory disease was completed. Admission was based on clinical criteria of significant leukocytosis, fever, suspected tubo-ovarian abscess (TOA), or recent outpatient treatment failure that were felt to warrant parenteral antibiotic therapy. The patients were prescribed one of three treatment options including Gentamicin/Clindamycin (with or without Ampicillin) and Cefotetan/Doxcycline. The patients were evaluated daily by a physician with treatment failures determined by failure to respond to the initial antibiotic regimen or clinical deterioration. Treatment failures had alternative antibiotic regimens completed. A formal cost analysis of the treatment options was completed.
**Results:** The treatment outcome for patients with pelvic inflammatory disease without tubo-ovarian abscess was found to be similar with either antibiotic treatment option. However, the daily cost of the chosen antibiotic therapy varied significantly. Treatment failures were most strongly associated with the presence of TOA. Patients with TOA were found to have a significantly shorter hospital stay when treated with Ampicillin/Gentamicin/Clindamycin when compared with Gentamicin/ Clindamycin alone or Cefotetan/ Doxcycline.
**Conclusion:** As concerns for cost-effective inpatient clinical care escalate, given equivalent outcomes for treatment of PID without TOA, the most cost effective antibiotic regimens should be chosen. In patients with TOA, triple therapy with Ampicillin/Gentamicin/ Clindamycin proved to be more effective and therefore more cost-effective for treatment.

**COST EFFECTIVENESS OF DOXYCYCLINE VERSUS AZITHROMYCIN IN THE TREATMENT OF *CHLAMYDIA TRACHOMATIS* INFECTIONS OF THE CERVIX.** Ransom, SB, McNeeley SG, Mazzoni M, Hendrix S. Division of Gynecology, Wayne State University School of Medicine, Detroit, MI

**Objective:** As concerns of cost effective treatment gains importance, clinical providers must be knowledgeable regarding the most clinically effective treatment modality for common disorders. This research was designed to compare the cost-effectiveness of doxycycline versus azithromycin in the treatment of chlamydial infections of the cervix.
**Study Design:** Sixty consecutive patients with *Chlamydia trachomatis* genital infections were randomly assigned to treatment with azithromycin, one gram po or doxycycline, 100mg BID for seven days. Study patients were aged 18–30 years without any other medical problems. Patients received outpatient therapy and returned 3 weeks after completion for re-evaluation. During the treatment period through re-evaluation, the patients refrained from coitus, avoided alcohol and drugs, and all other medications. The patients were tested with a DNA probe for *Chlamydia trachomatis*. The patients were randomized by a nurse and were blinded for study purposes to the evaluating physician.

**Results:** Chlamydial infection cure rates were not significantly different between the two groups [azithromycin (29/30) and doxycycline (25/30)]. No significant complications were found in either treatment group. The complete clinic cost for treatment was $26.15 for azithromycin and $6.50 for doxycycline.

**Conclusion:** The most cost-effective treatment for genital chlamydial infections in this small study appeared to be doxycycline. However, while treatment with azithromycin was significantly more expensive than doxycycline, there were slightly fewer treatment failures.

### CHLAMYDIA TRACHOMATIS IN PREGNANCY: PREVALENCE, RISK FACTORS, AND PREGNANCY OUTCOME.

Renee Fogelberg, MD, David Robin Field MD, Ira Golditch MD, Howard Barkin, Ph.D.

**Objectives:** This study looks at the prevalence of chlamydia in the population of pregnant females who receive prenatal care and deliver at Kaiser Permanente Medical Center, San Francisco. Risk factors for positive testing are outlined. Specific risk factors analyzed include: age, ethnicity, maternal occupation, marital status, and past history of sexually transmitted disease. Pregnancy outcomes (gestational age, weight, and infectious morbidities) related to positive cultures are profiled.

**Study Design:** A retrospective population based cohort study of 1255 women who received a chlamydia screen in their pregnancy, between November 1994 and June 1996. Epidemiologic and pregnancy outcome data are collected by chart review. Positive screens are detected by enzyme-immunoassay (EIA) based methods.

**Results:** Chlamydia is identified in less than 5% of this patient population. 68% of patients have a single screening test. No statistically significant difference is noted in poor pregnancy outcomes (low birth weight, and gestation age less than 37 weeks) for those patients who are screen-positive for chlamydia.

**Conclusion:** This study reflects a unique population that have most of their screening tests during the first trimester. The low prevalence rate of disease makes it difficult to note a significant impact on pregnancy outcome. It is not known whether these infections represent a chronic disease state, which might have less effect on a given pregnancy. The inherent limitations of a retrospective, non-case controlled study, which did not control for other infectious pathogens is noted.

**References:** Gravett MG, Nelson HP, et al: Independent association of bacterial vaginosis and chlamydia trachomatis infection with adverse pregnancy outcome. JAMA 1986; 256:1899–1903.

Sweet RL, Landers DV: Chlamydia trachomatis infection and pregnancy outcome. AM J Obstet Gynecol 1987; 156:824–33.

### EXPERIMENTAL MODEL OF NEONATAL GONOCOCCAL INFECTION, TRANSMISSION OF *N. GONORRHOEAE* FROM PREGNANT MOTHER TO THE FETUS IS C1q DEPENDENT.

Stella Nowicki, D.D.S.[1,2] and Bogdan Nowicki, M.D., Ph.D.[1,2]. Departments of Ob/Gyn[1], & Microbiology[2], University of Texas Medical Branch, Galveston, Texas.

Gonorrhea in pregnant women is associated with increased risks of spontaneous abortion and perinatal infant mortality, but is uncertain whether gonococcal infection is directly responsible for these complications or is a marker of high risk pregnancy due to other pathogenic mechanisms.

**Objectives:** The purpose of this investigation was to evaluate the effects of gonococcal infection during pregnancy on neonatal outcomes.

**Study Design:** Rats in day 20 of pregnancy were infected by intraperitoneal inoculation (i.p.) with three different *N. gonorrhoeae* strains originating from patients with: pelvic inflammatory disease (PID), disseminated gonococcal infection (DGI) and local infection (LI). Each group was divided into two subgroups: inoculated with $5 \times 10^7$ *N. gonorrhoeae* pretreated with C1q or without C1q. The study included 65 pregnant rats total. Blood samples were collected from pregnant and neonatal rats 48 h postinoculation and cultured on modified Thayer-Martin agar plates in triplicate for 24h in $CO_2$ incubator.

**Results:** The blood cultures of all 60 pregnant rats were negative. Interestingly, blood cultures of neonates were positive in two subgroups inoculated with PID and DGI strain pretreated with human C1q, but not in groups without C1q treatment. Results showed that experimental infection during pregnancy with PID and DGI but not LI strains of *N. gonorrhoeae* resulted in developing neonatal bacteremia in the presence of human factor C1q.

**Conclusions:** Neonatal gonococcal infection was highly correlated with perinatal infant morbidity (68%) and mortality (30%). We conclude that C1q dependent experimental gonococcal infection of pregnant rats may be relevant for evaluation of pathogenesis of neonatal bacteremia and associated complications.

### COST EFFECTIVENESS EVALUATION OF METRONIDAZOLE VERSES METROGEL VAGINAL® IN THE TREATMENT OF BACTERIAL VAGINOSIS.

Ransom, SB, McNeeley SG, Mazzoni M, Hendrix S. Division of Gynecology, Wayne State University School of Medicine, Detroit, MI

**Objective:** As managed care and concern of cost effectiveness expands, clinical providers must be knowledgeable regarding the most clinically efficient treatment modality for common disorders. This research was designed to compare the cost-effectiveness of metronidazole verses Metrogel Vaginal® in the treatment of bacterial vaginosis.

**Study Design:** 60 consecutive patients with the clinical diagnosis of bacterial vaginosis were randomly assigned prospectively into either a metronidazole (500mg BID × 7d) or Metrogel Vaginal® (one applicator BIS × 5d) treatment group. The study patients were aged 18–30 years without any other medical problems. The patients proceeded with outpatient therapy and returned 7–10 days after the completion of treatment for reevaluation. During the study, the patients refrained from sexual relations, avoided alcohol and drugs, and avoided all other medications. The physician evaluated the patients for bacterial vaginosis through standard wet prep, whiff test and pH testing prior to and after treatment. The patients were randomized by a nurse and were blinded for study purposes to the evaluating physician.

**Results:** The successful treatment outcomes for bacterial vaginosis was found to be 27 and 28 patients for Metrogel Vaginal® and metronidazole, respectively, out of the original 30 patients in each study group. All patients introduced into the study completed the study without difficulty. No significant complications were found in either treatment group. Three patients treated with metronidazole experienced Nausea during the treatment interval. The entire cost for treatment was \$19.71 and \$1.51 for Metrogel Vaginal® and metronidazole, respectively.

**Conclusion:** The most cost-effective treatment for bacterial vaginosis was found to be generic metronidazole. While the use of the more expensive Metrogel Vaginal® may be reasonable for patients experiencing side effects of metronidazole, most patients should be treated with the less expensive metronidazole.

### INTEGRATION OF IN VITRO SUSCEPTIBILITY MIC ACTIVITY OF TROVAFLOXACIN AGAINST ANAEROBES WITH HUMAN PHARMACOKINETIC DATA.

Kenneth E. Aldridge, Ph.D

Trovafloxacin is a new fluoroquinolone with broad-spectrum antibacterial activity which includes aerobic gram-positive cocci and anaerobes. Animal models indicate that there is a positive correlation between the bacteriologic eradication of organisms and the ratio of antibiotic concentration to in vitro MIC values. This study integrated in vitro susceptibility data of trovafloxacin against anaerobes with pharmacokinetic data to give trafloxacin: MIC ratios and to determine the time the trovafloxacin concentration was above the MIC. Comparisons were based on a 300 mg dose of trovafloxacin which yielded a plasma Cmax-4.3 μg/ml which was divided by the mode MIC (T/mm) and $MIC_{90}$ (T/M90). Time above the MIC's (hrs: T > MM; T > M90 was determined from the plasma profile of trovafloxacin over 48 hrs. Among the various species of the *B. fragilis* group the T/MM ranged from 8.6 to 34.4 and the T > MM ranged from 29 to 36 hrs. With the exception of *B. vulgatus* the T/M90 ranged from 4.3 to 8.6 and the T > $MIC_{90}$ ranged from 12 to 29 hrs. For other anaerobic gram-negative bacilli (*Prevotella, Porphyromonas, Fusobacterium*) the values were T/MM-8.6 to 17.2, T > MM-29 to 36 hrs, T/$MIC_{90}$-4.3 to 8.6, and T > M90-4.3 to 8.6 hrs. For isolates of *Clostridium, Eubacterium, Peptostreptococcus,* and *Veillomella* the values were T/MM-34.4 to 137.6, T > MM- > 36 hr., T/M90-8.6 to 17.2, and T > M90-29 to > 36 hrs. These data indicate that a single 300 mg dose of trovafloxacin produces favorable ratios to predict efficacy against these clinically significant pathogens. Moreover, trovafloxacin is highly tissue concentrated and when these data are available for the uterus and vagina ratios are also expected to be highly favorable.
